# Evaluation of Pediatric Manual Wheelchair Mobility Using Advanced Biomechanical Methods

**DOI:** 10.1155/2015/634768

**Published:** 2015-02-23

**Authors:** Brooke A. Slavens, Alyssa J. Schnorenberg, Christine M. Aurit, Adam Graf, Joseph J. Krzak, Kathryn Reiners, Lawrence C. Vogel, Gerald F. Harris

**Affiliations:** ^1^Department of Occupational Science & Technology, University of Wisconsin-Milwaukee, Milwaukee, WI 53201, USA; ^2^Rehabilitation Research Design and Disability (R_2_D_2_) Center, University of Wisconsin-Milwaukee, Milwaukee, WI 53201, USA; ^3^Orthopaedic and Rehabilitation Engineering Center (OREC), Marquette University and Medical College of Wisconsin, Milwaukee, WI 53233, USA; ^4^Shriners Hospitals for Children—Chicago, Chicago, IL 60707, USA; ^5^Physical Therapy Program, College of Health Sciences, Midwestern University, Downers Grove, IL 60515, USA

## Abstract

There is minimal research of upper extremity joint dynamics during pediatric wheelchair mobility despite the large number of children using manual wheelchairs. Special concern arises with the pediatric population, particularly in regard to the longer duration of wheelchair use, joint integrity, participation and community integration, and transitional care into adulthood. This study seeks to provide evaluation methods for characterizing the biomechanics of wheelchair use by children with spinal cord injury (SCI). Twelve subjects with SCI underwent motion analysis while they propelled their wheelchair at a self-selected speed and propulsion pattern. Upper extremity joint kinematics, forces, and moments were computed using inverse dynamics methods with our custom model. The glenohumeral joint displayed the largest average range of motion (ROM) at 47.1° in the sagittal plane and the largest average superiorly and anteriorly directed joint forces of 6.1% BW and 6.5% BW, respectively. The largest joint moments were 1.4% body weight times height (BW × *H*) of elbow flexion and 1.2% BW × *H* of glenohumeral joint extension. Pediatric manual wheelchair users demonstrating these high joint demands may be at risk for pain and upper limb injuries. These evaluation methods may be a useful tool for clinicians and therapists for pediatric wheelchair prescription and training.

## 1. Introduction

Among children under the age of 18, the wheelchair is the most widely used assistive mobility device impacting over 88,000 children, 90% of which use manual wheelchairs [1]. According to the 2012 Americans with Disabilities Report, approximately 3.7 million people use a wheelchair, with around 124,000 wheelchair users under the age of 21 and 67,000 under the age of 15 [2]. Despite this large number of pediatric wheelchair users, there is very limited information quantifying pediatric wheelchair mobility. This research will be valuable to the field of pediatric rehabilitation for improving clinical care to a developing and growing population of children and adolescents with spinal cord injury (SCI) and other orthopaedic disabilities. While a larger body of knowledge exists surrounding adult wheelchair biomechanics, it should not be assumed that the developing child experiences the same loading demands or is exposed to similar risk factors for overuse injuries, particularly during maturation. It is clear that pediatric onset SCI affects all aspects of quality of life, including mobility, participation, and function [3]; however, the impact of pediatric onset SCI on wheelchair biomechanics and the development of UE pain and pathology is unclear. Furthermore, it is unknown how each of the upper extremity joints (e.g., shoulder complex, elbow, and wrist) is affected during wheelchair mobility.

SCI is one of the leading causes of wheelchair usage in children and adolescents. The estimated annual incidence of SCI in the United States is approximately 40 cases per million population or approximately 12,000 new cases each year [4]. There are approximately 273,000 persons with SCI in the United States [4], and this number is rising, the majority of which heavily relies upon manual wheelchairs for mobility and community participation. Manual wheelchair use requires the upper extremities for mobility, performing transfers and weight relief, and performing activities of daily living (ADLs). The upper extremity is not intended for this load magnitude or frequency. Adult manual wheelchair users with SCI have a reported prevalence of 1/3 to 1/2 with upper extremity pain and/or deterioration of function and injury, including destructive shoulder arthropathy, degenerative arthritis of the shoulder and elbow, rotator cuff tendonitis, coracoacromial pathology, and carpal tunnel syndrome [5–11]. More specifically, estimates of shoulder pain among manual wheelchair users with paraplegia range from 30% to 73% [6, 7, 10, 12, 13]. It has been shown that shoulder pain and degenerative changes, especially at the acromioclavicular joint, may develop prematurely in individuals with SCI due to overuse and altered mechanical stresses, particularly in those with high levels of manual wheelchair activity [5, 6]. It has also been determined that manual wheelchair users who propel at a faster cadence and experience greater peak resultant handrim forces relative to body weight also have greater incidence of impaired median nerve function [13, 14]. Due to longer-term wheelchair use from pediatric onset SCI, these injuries may occur earlier and reduce or severely limit independence, function, and quality of life in children. Further insight into wheelchair mobility biomechanics of pediatric wheelchair users is critical for ultimately preventing these complications and improving their quality of life.

Upper limb pain and pathologies have been associated with increased loading at extremes of joint excursions [15]. Manual wheelchair use in adults has shown high shoulder joint loading with forces ranging from 7% to 12% of body weight, of which our prior study of a child with SCI agrees [6, 16, 17]. High joint forces during manual wheelchair use have been shown to directly correlate to the cause of joint pain and injury [6]. Additionally, previous research has also investigated wheelchair stroke patterns in adults, the motion the hand makes during the stroke cycle, which only differentiates during the recovery phase, when the hand is not in contact with the handrim and is restricted to its movement. Four different propulsion patterns have been identified in adult users, by the hand's motion during the recovery phase: single-looping over propulsion (when the hands rise above the handrim), double-looping over propulsion (when the hands rise above and then fall below the handrim), semicircular (when the hands fall below the handrim), and arcing (when the hand follows the path of the pushrim). This research also showed that in adults, the semicircular pattern resulted in the lowest cadence to go the same speed and the greatest percentage of time spent in the contact phase, allowing the user to impart force to the handrim over a greater angle and longer time. Since both of these parameters have been linked to reduction of injury in adults, the semicircular pattern is the recommended technique for adult manual wheelchair propulsion [12, 13, 18]. A study by Mercer et al. supports further investigation of wheelchair design, prescription, training, and propulsion biomechanics that reduce shoulder joint forces and moments to preserve upper limb integrity [6]. Quantification of 3D joint dynamics in children is essential for uncovering the root of secondary injuries. In order to evaluate the pediatric population of manual wheelchair users, it is essential that biomechanical methods are applied that consider the unique musculoskeletal features of a child.

This study seeks to address the knowledge gap in biomechanical evaluation of pediatric wheelchair mobility by quantifying and characterizing upper extremity joint dynamics during manual wheelchair mobility. Specifically, we will quantify shoulder complex, elbow, and wrist joint demands during mobility to determine the ranges of motion, forces, and moments, which may play a key role in the clinical evaluation of joint integrity and wheelchair skills and performance. We hypothesize that proximal joint ranges of motion, joint forces, and joint moments will be significantly different than distal joint dynamics. This work presents biomechanical methods for pediatric evaluation that aim to minimize the risk of developing secondary complications associated with wheelchair use, such as upper extremity pain and overuse related upper limb injuries.

This work is the first of its kind to quantify wheelchair biomechanics in children. Better knowledge of how to evaluate UE dynamics during wheelchair propulsion may enhance our understanding of the onset and propagation of UE pain and secondary pathologies. This may lead to improvements in wheelchair prescription, design, training, and transitional care. Ultimately, the incidence of manual wheelchair use related pain and pathology might be reduced while maximizing function and quality of life of children with SCI.

## 2. Materials and Methods

### 2.1. Subjects

Twelve pediatric manual wheelchair users with spinal cord injury were evaluated at Shriners Hospitals for Children—Chicago. Subject characteristics are described in Table 1. IRB approval was obtained and an assent form or informed consent form was signed by the child and/or their parent/guardian. Subject inclusion criteria: under 21 years of age, chronic incomplete SCI diagnosis at least one year after injury, and manual wheelchair as primary mode of mobility.

### 2.2. Data Collection

Subject specific measurements were obtained and twenty-seven passive reflective markers were placed on bony anatomical landmarks and technical locations of the subject, including the suprasternal notch, xiphoid process, spinal process C7, acromioclavicular joint, inferior angle, trigonum spinae, scapular spine, acromial angle, coracoid process, humerus technical marker, olecranon, radial and ulnar styloids, and the third and fifth metacarpals [17]. Based on validated methods by Šenk and Chèze, due to the subcutaneous motion of the scapula, the trigonum spinae and inferior angle markers were only used during a static trial with the subject in anatomical position. During dynamic trials, the positions of these markers were calculated using a method based on rigid body theory, shown to be appropriate for scapular motion tracking especially during tasks with less than 120 to 150 degrees of arm elevation. This method had low RMS errors (5.4–10.3 deg), similar to those of commonly used tracker (3.2–10.0 deg) and acromion (4.8–11.4 deg) methods [19]. A SmartWheel (Outfront, Mesa, AZ), with an air tire, replaced the wheel on the dominant side of the subject's wheelchair for kinetic data collection; the SmartWheel companion wheel replaced the subject's wheel on the nondominant side. Plastic coated handrim attachments were not used on either wheel for any of the subjects as none of the subjects used coated handrims or gloves to assist with their propulsion.

**Figure 1:**
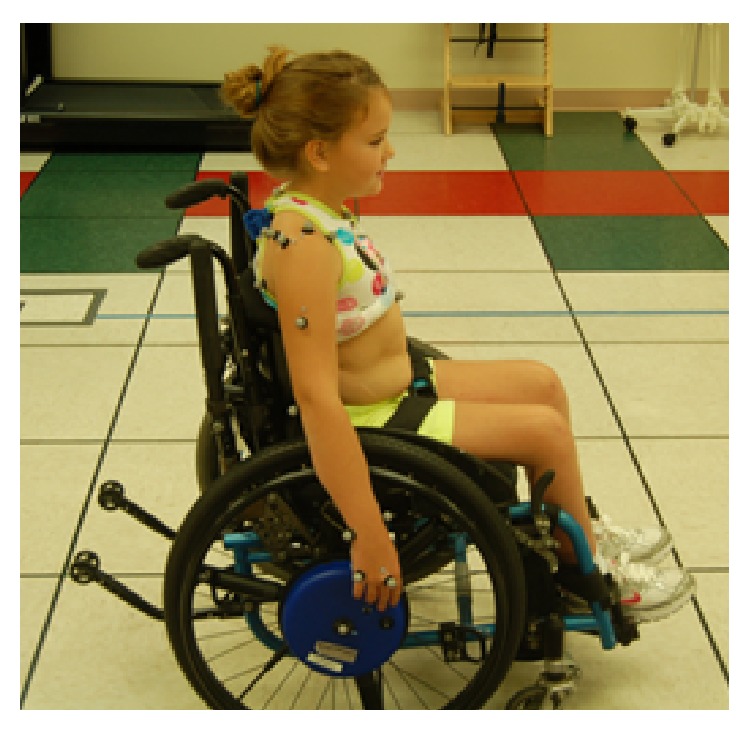
Subject preparing to begin motion analysis with the SmartWheel.

The subject propelled his or her manual wheelchair along a 15 m walkway at a self-selected speed and self-selected propulsion pattern (Figure 1). A 10-camera Vicon MX system captured the 3D marker trajectories at 120 Hz, while simultaneously the SmartWheel collected the 3D forces and moments occurring at the hand-handrim interface at 240 Hz. Multiple trials were collected, with adequate rest provided to the subject as needed.

### 2.3. Upper Extremity Biomechanical Model

A custom bilateral pediatric upper extremity model was applied to the data to determine 3D joint angles, forces, and moments [17]. This biomechanical model comprises 11 segments, including thorax, clavicles, scapulae, upper arms, forearms, and hands. The joints of interest are three-degree-of-freedom thorax, wrist, glenohumeral, and acromioclavicular joints and two-degree-of-freedom sternoclavicular and elbow joints.

Segment coordinate systems (SCS) were determined for each of the model's 11 segments. Following ISB recommendations, the SCS axes are aligned such that the *Z*-axis points laterally towards the subject's right side, the *X*-axis points anteriorly, and the *Y*-axis points superiorly [20]. The joint angles were determined by the relative motion between two adjacent SCS, distal relative to proximal. A* Z*-*X*-*Y* Euler sequence is used to calculate the glenohumeral, elbow, wrist, and thorax joint angles, and a* Y*-*X*-*Z* Euler sequence is used for the acromioclavicular and sternoclavicular joint angle computation. The SCS follow the right-hand rule with the *Z*-axis as the flexion-extension axis, the *X*-axis as the abduction/adduction axis, and the *Y*-axis as the internal-external rotation axis.

### 2.4. Data Processing

Vicon Nexus was used to process the marker trajectories. Any gaps in the data were filled using the cubic spline interpolation feature and the resulting marker trajectories were filtered using a Woltring filter with a mean squared error setting of 20. The kinetic data from the SmartWheel was filtered using a low pass FIR filter. The kinetic data was then resampled to 120 Hz in MATLAB.

**Figure 2:**
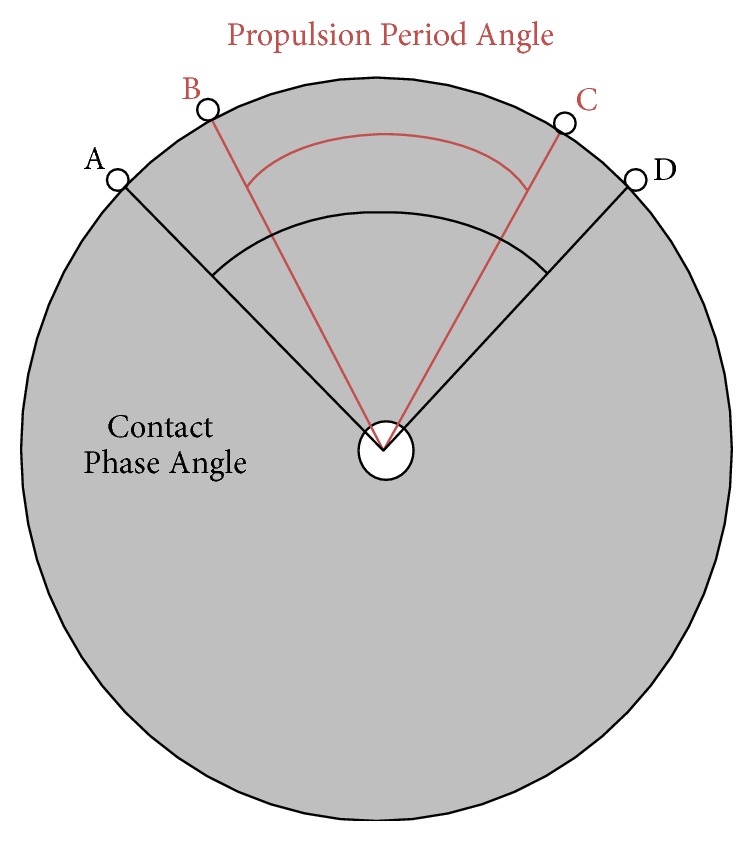
Contact phase angle and propulsion period angle. The contact phase angle begins at Point A, when a force is detected on the handrim, indicating hand contact, and ends at Point D, when a handrim force is no longer present. The propulsion period angle begins at Point B, when a propulsive moment about the wheel axle is present, and ends at Point C, when the propulsive moment is no longer detected.

For each subject, ten wheelchair stroke cycles, compiled from multiple trials, were analyzed to produce average subject parameters, which were then used to compute the average group parameters of interest. Only strokes occurring during steady-state propulsion were included in the analyses; the start-up and stopping pushes were excluded. Average time series data of the joint angles, forces, and moments were all time-normalized to percent of the wheelchair stroke cycle. The stroke cycles were separated into two phases (contact and recovery) based on total force applied to the handrim, with the contact phase subdivided into periods of propulsive contact (propulsion) and nonpropulsive contact (initial contact and release) as determined by the moment about the wheel axle [21]. Contact phase angle, the angle the wheel moved during hand contact with the handrim, was determined by subtracting the wheel angle at the end of the contact phase from the wheel angle at the start of contact phase. The propulsion period angle, the angle the wheel moved during propulsive hand contact, was similarly defined during the propulsion period. These definitions for wheelchair stroke cycle phases and periods, and the angles, follow those described by Kwarciak et al. [21] (Figure 2). The stroke pattern was determined using the sagittal plane motion of the marker on the third metacarpal, plotting the vertical position versus fore-aft position [12].

Peak joint angles and angular ranges of motion were identified and calculated. All force data were normalized to percent body weight (%BW) and all moment data were normalized to percent body weight times height (%BW ×* H*). Peak joint forces and moments were also identified. Two sample *t*-tests were used for statistical comparisons amongst group average joint ranges of motion and average peak dynamics.

## 3. Results

### 3.1. Temporal-Spatial Parameters

The average propulsion speed was 1.23 m/s ± 0.26 m/s, ranging from 0.79 m/s to 1.6 m/s, with an average cadence of 1.1 strokes/sec ±0.2 strokes/sec. The average contact phase occurred from 0% to 35.8% stroke cycle with a range of 25% to 45% stroke cycle. Within the contact phase, the initial contact period occurred on average from 0% to 3.6% stroke cycle, the propulsion period on average occurred from 3.6% to 34.1% stroke cycle, and the release period occurred on average from 34.1% to 35.8% stroke cycle. The average contact phase angle was 85.6° ± 15.7°. The average propulsion period angle was 72.6° ± 11.9°. Lastly, the average peak resultant handrim force was 10.1% BW ± 3.7% BW.

Additionally, there was a wide array of propulsion patterns utilized by the children. One subject used the single-looping overpropulsion (SLOP) pattern, 3 subjects used the double-looping overpropulsion (DLOP) pattern, and 3 subjects used the recommended semicircular (SC) pattern [12]. The remaining five subjects used a mixture of patterns making the primary pattern unidentifiable.

### 3.2. Joint Kinematics

**Figure 3:**
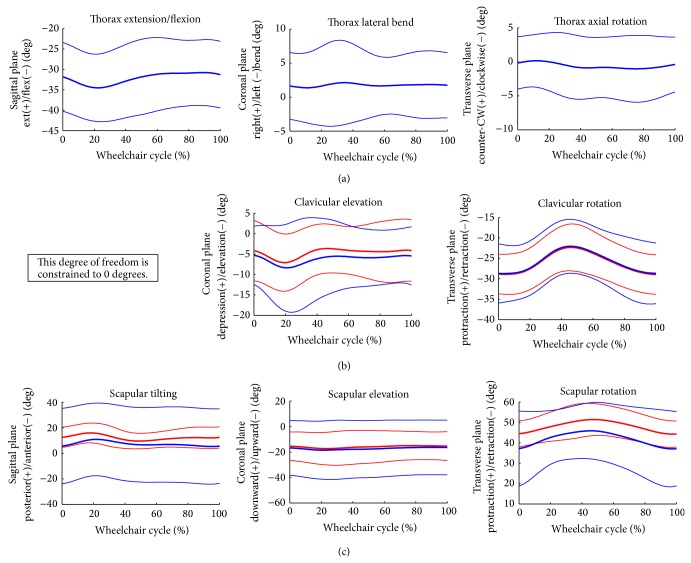
Group average joint kinematic data of the thorax, sternoclavicular, and acromioclavicular joints. Mean (bold) and +/− one standard deviation joint kinematics of the thorax: top row, the sternoclavicular (SC) joint: middle row, and the acromioclavicular (AC) joint: bottom row (dominant side: blue, nondominant side: red).

**Figure 4:**
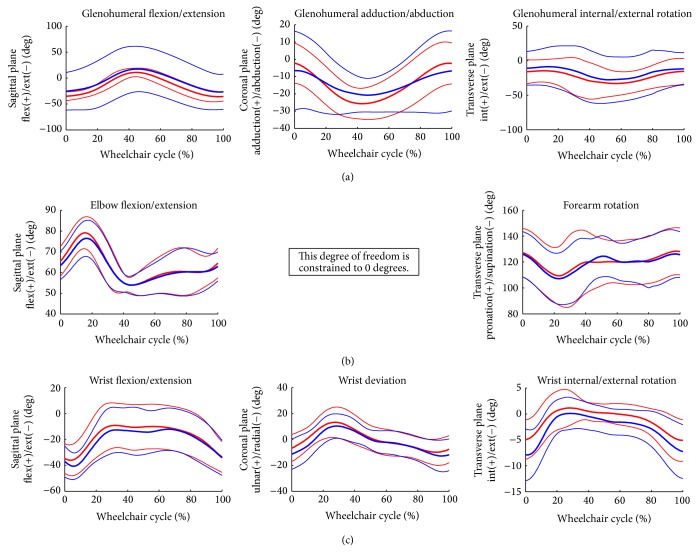
Group average joint kinematic data of the glenohumeral, elbow, and wrist joints. Mean (bold) and +/− one standard deviation joint kinematics of the glenohumeral (GH) joint: top row, the elbow joint: middle row, and the wrist joint: bottom row (dominant side: blue, nondominant side: red).

**Figure 5:**
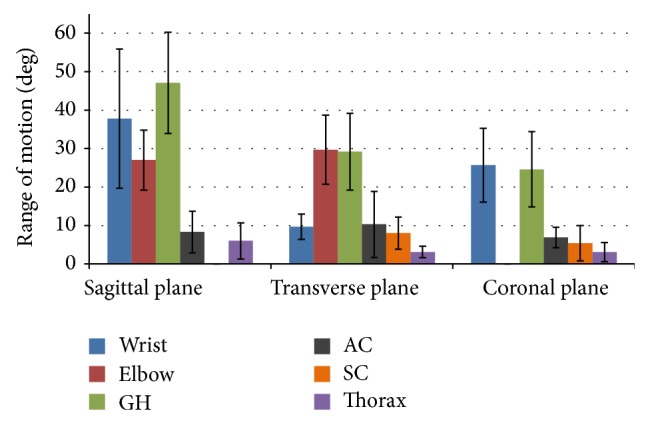
Group average joint ranges of motion (and standard deviation bars) for the thorax, sternoclavicular (SC), acromioclavicular (AC), glenohumeral (GH), elbow, and wrist joints in the sagittal, transverse, and coronal planes.

Group mean joint angles of the thorax, sternoclavicular, acromioclavicular, glenohumeral, elbow, and wrist joints were characterized over the wheelchair stroke cycle. These mean joint angles in each plane of motion, along with +/− one standard deviation, are depicted in Figures 3 and 4. The mean peak angles and ranges of motion (ROMs) of each joint were also identified and computed over the stroke cycle. The joint ROMs are depicted in Figure 5. Additionally, multiple parameters were statistically significantly different (*P* < 0.05) from one another, as seen in Table 2.

### 3.3. Joint Kinetics

**Figure 6:**
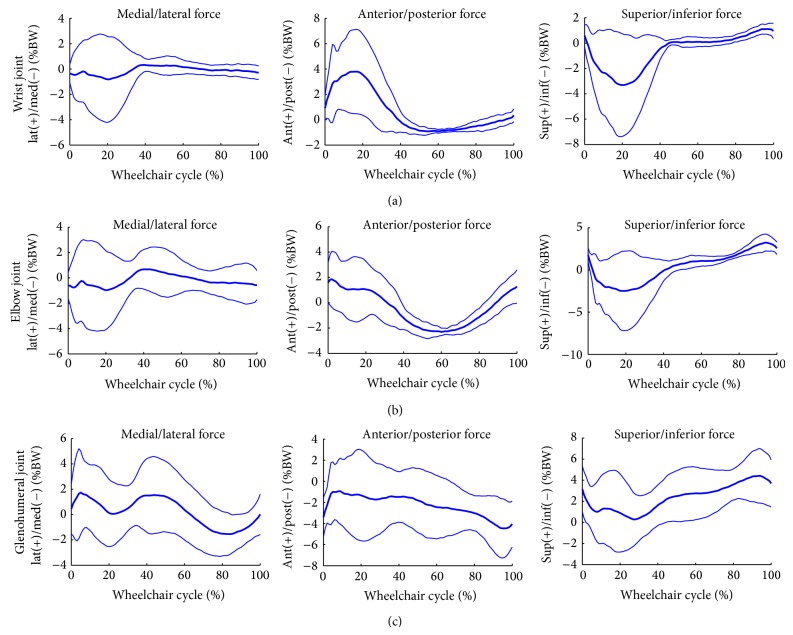
Group average joint force data of the dominant side wrist, elbow, and glenohumeral joints. Mean (bold) and +/− one standard deviation joint forces of the wrist joint: top row, elbow joint: middle row, and the glenohumeral (GH) joint: bottom row of the subjects' dominant side.

**Figure 7:**
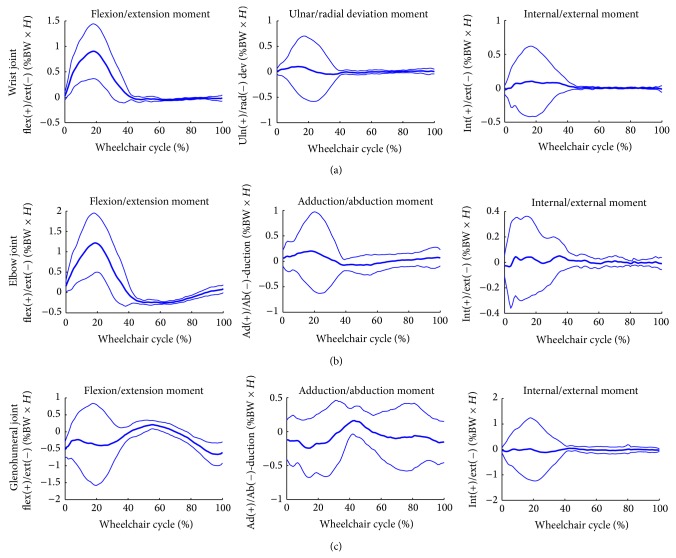
Group average joint moment data of the dominant side wrist, elbow, and glenohumeral joints. Mean (bold) and +/− one standard deviation joint moments of the wrist joint: top row, elbow joint: middle row, and the glenohumeral (GH) joint: bottom row of the subjects' dominant side.

Group mean joint forces and moments of the glenohumeral, elbow, and wrist joints were characterized over the wheelchair stroke cycle. The group's mean joint forces (+/− one standard deviation) along each axis are depicted in Figure 6. The mean group joint moments (+/− one standard deviation) in each plane of motion are depicted in Figure 7.

**Figure 8:**
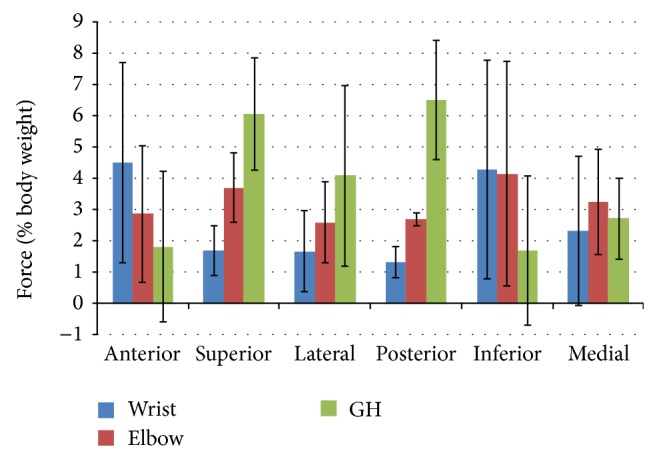
Group average peak joint forces (and standard deviation bars) of the subjects' dominant side glenohumeral, elbow, and wrist joints along the anterior-posterior, superior-inferior, and lateral-medial axes.

**Figure 9:**
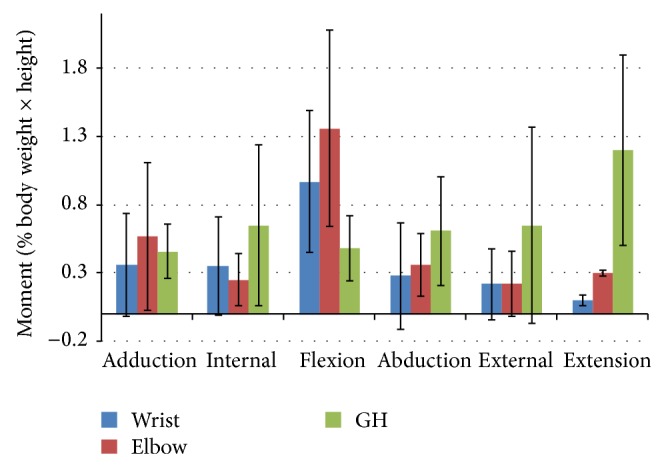
Group average peak joint moments (and standard deviation bars) of the subjects' dominant side glenohumeral, elbow, and wrist joints for adduction-abduction, internal-external, and flexion-extension rotations.

Additionally, average peak joint forces and moments were identified and shown in Figures 8 and 9, respectively. Statistically significant differences (*P* < 0.05) were found between the multiple parameters, as seen in Table 3. The glenohumeral joint forces were statistically significantly higher than the wrist joint forces directed superiorly, laterally, and posteriorly. The wrist joint forces in the anterior and inferior directions were significantly greater than those at the GH joint. Additionally, the GH joint experienced significantly higher joint forces directed superiorly and posteriorly than the elbow joint, while the elbow joint experienced significantly higher inferiorly directed force than the GH joint. Lastly, the elbow joint experienced significantly higher forces than the wrist in the superior, lateral, and posterior directions. When evaluating joint moments, the GH joint experienced significantly greater moments in flexion, abduction, external rotation, and extension than the wrist joint, while the elbow was only significantly greater than the wrist in the extension moment. Additionally, the GH joint experienced significantly higher moments than the elbow joint in internal rotation, abduction, external rotation, and extension. Both the elbow and wrist joints experienced significantly higher flexion moments than the GH joint.

## 4. Discussion

Our novel biomechanical model defined the UE, including the thorax, shoulder complex (humerus, scapula, and clavicle segments), elbow, and wrist for a comprehensive bilateral characterization [17]. The goal of this work was to quantify pediatric wheelchair mobility using new modeling techniques and evaluation approaches for the pediatric and young adult user.

The average self-selected speed of the children and young adults (1.23 m/s) was comparable to the self-selected speed of adult manual wheelchair users with SCI (1.07 m/s) in a study by Collinger et al. [16]. While self-selected speeds are important for evaluating typical, everyday activity, future work investigating pediatric propulsion should also consider assessing steady-state target speeds since biomechanical parameters may vary with speed [16].

The temporal-spatial parameters were compared with two other studies involving adult manual wheelchair users with SCI (Table 4) [22, 23]. The children and young adults in this study exhibited on average low cadences, which are recommended for injury prevention. However, they also experienced peak resultant, weight-normalized, handrim force similar to those of adult manual wheelchair users. While these force levels have been correlated to long-term pain and pathology in adult users, it is still unknown what effect similar loading would have on pediatric and young adult users. Additionally, while the average propulsion period angles were similar between this study and that of Gil-Agudo et al. (where this term is called “propulsion angle”) and Kwarciak et al., the contact phase angles appear to be much smaller among pediatric and young adult users than the adults in the Boninger et al. (where this term is called “push angles”) and Kwarciak et al. studies. Further evaluation is required to determine if smaller stature, greater efficiency, or other factors are the reason for this difference. Inconsistencies among cadence values as they relate to velocity, as our subjects had higher speed than the adults investigated in Gil-Agudo et al.'s study with lower cadence, further indicate a need to investigate temporal-spatial parameters of children and young adult manual wheelchair users [22, 23]. The correlation among these parameters, as well as biomechanical metrics, to other factors of clinical history, such as age, diagnosis and injury level, time since injury, and time of manual wheelchair use, may be particularly insightful.

The average relative time spent in the contact phase of the stroke cycle (35.8%) fell within the range commonly reported for adult manual wheelchair users of 30% to 50% [24]. It has been shown that increased relative time of the contact phase is indicative of more challenging tasks, such as propelling with increased resistance or up a ramp [24], and while the propulsion evaluated here was not considered challenging, there were a few subjects whose relative time in the contact phase was around 45%. Perhaps this measure is indicating that this task was more demanding for these children. Likewise, we found some children whose relative time in contact phase was around 25%, slightly below the commonly reported range. As it is recommended to take long, smooth propulsive strokes [13], this shortened contact time with the handrim could result in higher force and rate of force application and thus be related to higher joint demands. Additionally, the model captured times of nonpropulsive moments on the handrim, indicating a braking effect, or nonefficient movements. The presented biomechanical methods may be used for evaluation of efficiency and training.

Propulsion patterns used by the pediatric and young adult population in this study were varied and subjects often either switched patterns between trials or did not clearly use one of the four common patterns described in the literature [12, 18], thus making identification of the primary pattern difficult. While the semicircular propulsion pattern is the recommended pattern for reduced joint loading and cadence [13], it has not yet been shown if this pattern is appropriate for the pediatric population. The semicircular pattern may not be appropriate or attainable for the pediatric propulsion due to physical limitations, wheelchair prescription or set-up (either originally or due to the growth of the child), or improper training. Given the patterns observed in this study, further investigation is warranted on appropriate propulsion patterns in the pediatric population of manual wheelchair users.

Overall, joint ranges of motion ranged from 3.1° to 47.1°, with the largest ROM at the glenohumeral joint during flexion/extension. Significant differences were found between joint ROMs of the glenohumeral, elbow, and wrist joints during internal-external rotation and flexion/extension. Peak joint forces ranged from 1.31% BW posteriorly at the wrist to 6.08% BW superiorly at the glenohumeral joint. Moments ranged from 0.1% BW ×* H* of wrist extension to 1.36% BW ×* H* of elbow flexion. These forces and moments are of concern in the pediatric population since they are similar to the magnitudes reported in adults [6, 23, 25].

These findings support continued quantitative evaluation of joint biomechanics for the prevention of pain and overuse injuries, of which these children may be at risk. The shoulder joint demonstrated the highest ROMs and forces compared to the distal elbow and wrist joints, as hypothesized. However, joint moments proved to be the highest at the elbow, followed by the glenohumeral joint and wrist joint. This may be due to mechanical inefficiency, lack of adequate training, and/or asymmetry. In addition, the variation of stroke patterns and speed may have impacted our group means. This further supports subject specific analyses in the future. This work has potential to be applied for in-depth quantitative evaluation of these factors. While much work has been conducted examining adult biomechanics during wheelchair use and the effects on upper limb injuries, particular consideration should be given to children. It is important that biomechanical evaluation methods are applied when prescribing, training, and providing long-term, transitional care of pediatric wheelchair users. Further work is underway to investigate the effects of age, duration of wheelchair use, muscular and soft tissue contributions, and level of injury. Ultimately, this work may lead to the development of guidelines for optimal pediatric wheelchair mobility.

## 5. Conclusions

This study presents findings for three-dimensional (3D) evaluation of joint dynamics of the shoulder complex, elbow, and wrist in children with SCI. Currently, pediatric wheelchair mobility biomechanics have not been reported. Evaluation methods for effective quantification of upper extremity joint dynamics during wheelchair propulsion have been presented. A population of 12 children with SCI was characterized. Joint ranges of motion and forces were found to be the greatest at the glenohumeral joint. Magnitude and frequency of joint demands are of concern for long-term manual wheelchair use in those with pediatric onset SCI. Parameters including range of motion, peak joint force, and peak joint moment should be assessed in 3D for all pediatric wheelchair users for rehabilitation planning. Further evaluation techniques and characterization should be investigated for prevention of pain and upper limb injuries. Particular attention should be given to this population of interest in regard to longer-term duration of wheelchair use and changes during development and maturation. Additional work is underway to correlate clinical history, pain, and functional outcomes to joint dynamics for further insight to provide patient-centered rehabilitation. Ultimately this work may lead to the development of pediatric guidelines for optimal wheelchair propulsion.

## Figures and Tables

**Table 1:** Subject characteristics for each subject and the calculated group averages and standard deviations.

Subject	Age (years)	Height (cm)	Weight (kg)	Gender	Arm dominance
1	9.8	137.1	31.9	Male	Right
2	7	121.9	26.5	Male	Left

3	8.7	128.0	28.2	Male	Right
4	20	167.6	51.1	Female	Left

5	18	129.5	61.2	Male	Right
6	18	177.8	54.0	Male	Left

7	17.7	67.8	49.0	Male	Left
8	16	170.2	63.1	Male	Right

9	11.3	154.2	34.7	Male	Right
10	17	151.3	41.3	Male	Left

11	8.6	124.4	31.7	Female	Right
12	6.5	119.3	28.5	Male	Right

Avg.	13.2	137.4	41.8		
St. dev.	5.0	29.9	13.4		

**Table 2:** *P* values for *t*-tests comparing all joint ranges of motion for each plane of motion. Italic cells indicate statistically significant differences (*P* < 0.05).

		Wrist	Elbow	GH	AC	SC
Elbow	Sagittal	0.076				
	Transverse	*<0.001 *				

GH	Sagittal	0.168	*<0.001 *			
	Transverse	*<0.001 *	0.899			
	Coronal	0.783	*N/A *			

AC	Sagittal	*<0.001 *	*<0.001 *	*<0.001 *		
	Transverse	0.834	*<0.001 *	*<0.001 *		
	Coronal	*<0.001 *	*N/A *	*<0.001 *		

SC	Transverse	0.270	*<0.001 *	*<0.001 *	0.411	
	Coronal	*<0.001 *	*N/A *	*<0.001 *	0.339	

Thorax	Sagittal	*<0.001 *	*<0.001 *	*<0.001 *	0.27514	
	Transverse	*<0.001 *	*<0.001 *	*<0.001 *	*0.015 *	*0.002 *
	Coronal	*<0.001 *	*N/A *	*<0.001 *	*0.002 *	0.164

**Table 3:** *P* values for *t*-tests comparing peak joint forces for all directions and *t*-tests comparing peak joint moments for all rotations. Italic cells indicate statistically significant differences (*P* < 0.05).

Force				Moment		
	Direction	Wrist	Elbow	Direction	Wrist	Elbow
Elbow	Anterior	0.161		Adduction	0.296	
	Superior	*<0.001 *		Internal	0.389	
	Lateral	0.092		Flexion	0.145	
	Posterior	*<0.001 *		Abduction	0.513	
	Inferior	0.921		External	0.980	
	Medial	0.289		Extension	*<0.001 *	

GH	Anterior	*0.033 *	0.279	Adduction	0.453	0.521
	Superior	*<0.001 *	*<0.001 *	Internal	0.153	*0.043 *
	Lateral	*0.019 *	0.128	Flexion	*0.009 *	*0.001 *
	Posterior	*<0.001 *	*<0.001 *	Abduction	0.052	0.085
	Inferior	*0.046 *	0.063	External	0.076	0.075
	Medial	0.629	0.402	Extension	*<0.001 *	*0.002 *

**Table 4:** Average (St. dev.) subject characteristics and temporal-spatial parameters (NR means not reported).

Parameter	Children and young adults		Adults			
	This study	Kwarciak et al., 2009 [21]	Boninger et al., 2000 [22]		Gil-Agudo et al., 2010 [23]	
Age (yrs)	13.2 (5)	40.7 (11.3)	35.2 (9.3)		37.5 (9.8)	
Height (cm)	137.4 (29.9)	NR	176 (9.9)		170 (10)	
Weight (kg)	41.8 (13.4)	76.6 (16.4)	75.3 (17.9)		70.1 (10.9)	
Diagnosis	SCI	SCI	SCI		SCI	

			Speed 1	Speed 2	Speed 1	Speed 2

Velocity (m/s)	1.2 (0.26)	1.08 (NR)	0.98 (0.13)	1.65 (0.18)	0.833 (NR)	1.11 (NR)
Cadence (stroke/sec)	1.05 (0.19)	NR	1 (0.2)	1.3 (0.3)	1.1 (0.2)	1.2 (0.3)
Contact phase angle (deg)	85.6 (15.7)	98.38 (NR)	100.9 (16.5)	110.7 (14.7)	NR	NR
Propulsion period angle (deg)	72.6 (11.9)	80.64 (15.68)	NR	NR	66.3 (16.5)	69.7 (16.8)
Peak resultant force (%BW)	10.1 (3.7)	NR	9.6 (3.5)	13.9 (4.5)	8.6 (2.1)	10.6 (4.1)
